# Field emission from *in situ*-grown vertically aligned SnO_2 _nanowire arrays

**DOI:** 10.1186/1556-276X-7-117

**Published:** 2012-02-13

**Authors:** Zhihua Zhou, Jiang Wu, Handong Li, Zhiming Wang

**Affiliations:** 1 State Key Laboratory of Electronic Thin Film and Integrated Devices, School of Microelectronics and Solid-State Electronics, University of Electronic Science and Technology of China, Chengdu 610054, China

## Abstract

Vertically aligned SnO_2 _nanowire arrays have been *in situ *fabricated on a silicon substrate via thermal evaporation method in the presence of a Pt catalyst. The field emission properties of the SnO_2 _nanowire arrays have been investigated. Low turn-on fields of 1.6 to 2.8 V/μm were obtained at anode-cathode separations of 100 to 200 μm. The current density fluctuation was lower than 5% during a 120-min stability test measured at a fixed applied electric field of 5 V/μm. The favorable field-emission performance indicates that the fabricated SnO_2 _nanowire arrays are promising candidates as field emitters.

## Introduction

SnO_2 _is a wide bandgap semiconductor (*E*_g _= 3.6 eV, 300 K) which has been widely applied in gas sensors, solar cells, lithium-ion batteries, and nanoelectronic devices [1-4] due to its outstanding optical and electrical properties [5]. Nanoscaled SnO_2 _presenting peculiar properties superior to its bulk counterpart because of the quantum effects has attracted much interest in recent years. Various methods have been developed to fabricate a SnO_2 _nanostructure, including thermal evaporation of the metal tin (Sn) [6], sonochemical method [7], carbothermal reduction [8], hydrothermal method [9], electrodeposition method [10], and so on. The field-emission [FE] properties of SnO_2 _nanobelts, nanoflowers, [11] and nanowires [12] were also studied considering the potential applications of FE flat displays, X-ray sources, and microwave devices. It was found that the nanowires and long nanobelts of SnO_2 _exhibited outstanding FE properties [13-15]. In general, vertically aligned nanowire arrays are the best candidates for FE sources because the efficiency of the field emitters is based on the extremely small radii of the tips, and the diameter of the nanowire, the precise position, and the alignment can be well controlled [16-18]. Therefore, it is necessary to develop a method to fabricate well-aligned SnO_2 _nanowire arrays to further improve the FE performance.

In this work, an *in situ *catalytic thermal evaporation method was developed to fabricate vertically aligned SnO_2 _nanowire arrays on a silicon substrate. The FE characteristics of the SnO_2 _nanowire arrays were studied. The *in situ*-grown SnO_2 _nanowire arrays, benefiting from the well-aligned structure and the *in situ*-grown fabrication method, demonstrated favorable low turn-on electric fields and a relatively stable emission behavior.

## Experimental details

For preparing the SnO_2 _nanowire arrays, 2 g of tin powder (Sinopharm Chemical Reagent Co., Ltd., Shanghai, China) was put in a ceramic boat. A silicon (100) substrate sputtered with a 5-nm-thick Pt film was placed on the top of the ceramic boat. The distance between the tin powder and the substrate was about 0.5 cm. The ceramic boat was placed in the middle of an electric resistance tube furnace. The electric resistance tube furnace was purged with a continuous 100-sccm high-purity nitrogen gas for 15 min beforehand. Then, it was heated up to 850°C at a rate of approximately 30°C/min and kept at 850°C for 10 min. Lastly, it was cooled down to room temperature naturally, and a white layer of product was found on the silicon substrate.

The surface morphology and crystal structure of the *in situ*-grown SnO_2 _nanowire arrays were investigated by scanning electron microscopy [SEM] (JEOL JEM-6320F, JEOL Ltd., Akishima, Tokyo, Japan), high-resolution transmission electron microscopy [HRTEM] (JEOL JEM-2100, JEOL Ltd., Akishima, Tokyo, Japan), and X-ray diffraction [XRD] (Bruker-AXS D8, Bruker Optik Gmbh, Ettlingen, Germany). The optical properties of the *in situ*-grown nanowire arrays were studied by Raman spectroscopy (French Labrum-HR cofocal laser micro-Raman spectrometer (Dilor S.A, Villeneuve d'Ascq, France) using an argon-ion laser at 514.5 nm).

The FE measurements were performed in a vacuum chamber at a pressure of 3 × 10^-5 ^Pa at room temperature. The silicon substrate with the *in situ*-grown SnO_2 _nanowire arrays served as the cathode, and a fluorine-tin-oxide coated glass served as the anode. The cathode and anode were separated with mica spacers. The applied electric field (*E*) was determined by dividing the applied voltage (*V*) by the anode-cathode separation (*d*). The emission density (*J*) was evaluated from the quotient of the obtained emission current divided by the cathode surface area (0.25 cm^2^).

## Results and discussion

**Figure 1:**
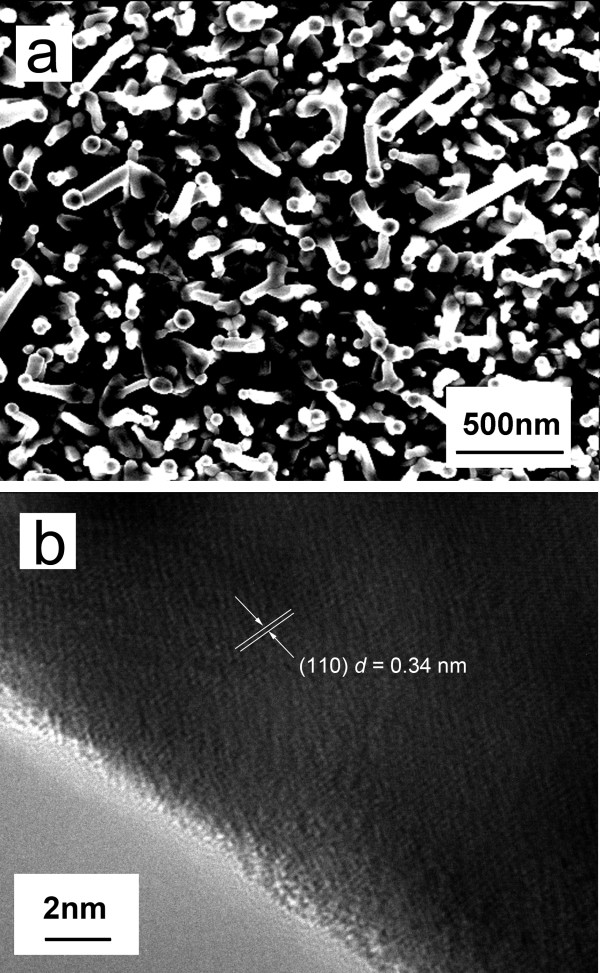
**Morphology characterization of the prepared SnO_2 _nanowire arrays**. A (**a**) typical SEM image and (**b**) HRTEM image.

Figure 1a shows a top view SEM image of the *in situ*-grown SnO_2 _nanowire arrays. It can be seen that the nanowire was vertically aligned on the silicon substrate with diameters between 40 to 60 nm. Noticeably, on the top of each nanowire, there was a globule, which strongly indicates that the nanowire grew via a vapor-liquid-solid mechanism [19]. The density of the SnO_2 _nanowire arrays, determined by counting nanowires in a representative area of a SEM image, was estimated to be 3 × 10^7^/mm^2^. Figure 1b presents a high-resolution TEM image of a single SnO_2 _nanowire. It shows that the SnO_2 _nanowire is a single crystalline nanowire with an interplanar spacing of 0.34 nm corresponding to the (110) plane of a rutile crystalline SnO_2_.

**Figure 2:**
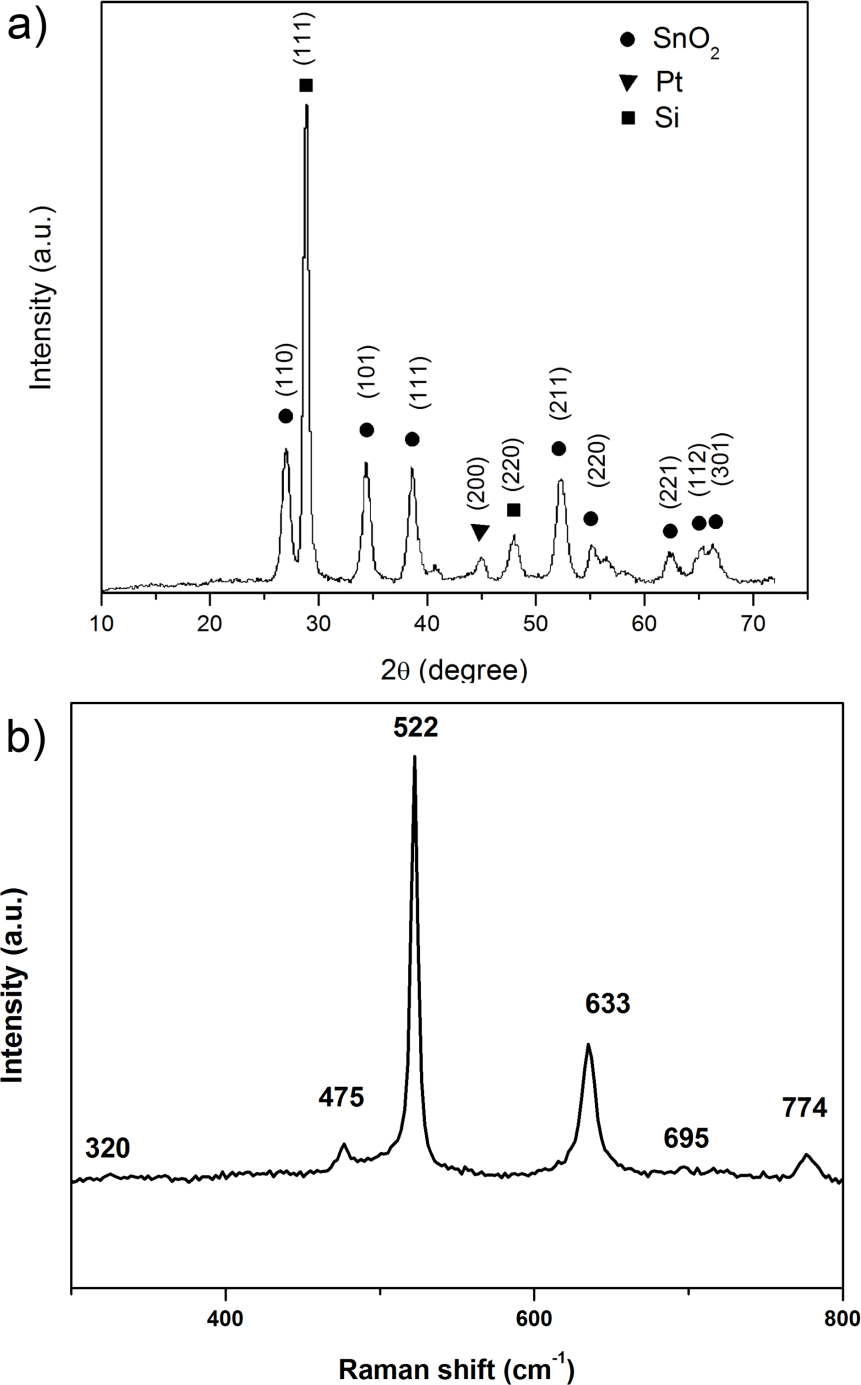
**X-ray diffraction pattern (a) and Raman spectrum (b) of the prepared SnO_2 _nanowire arrays**.

XRD characterization was employed to investigate the crystal structure of the *in situ*-grown SnO_2 _nanowire arrays. Figure 2a shows a typical XRD pattern. The diffraction peaks can be well indexed to the standard values of bulk SnO_2 _(JCPDS card: 41-1445), Si (JCPDS card: 27-1402), and Pt (JCPDS card: 88-2343). The peaks attributed to SnO_2 _demonstrate that the *in situ*-grown sample crystallized with the tetragonal rutile structure with lattice constants of *a *= 4.738 Å and *c *= 3.187 Å.

A typical room temperature Raman spectrum of the *in situ*-grown SnO_2 _nanowire arrays is shown in Figure 2b. It can be seen that there were three fundamental Raman peaks located at 475, 633, and 774 cm^-1^, which correspond to *E_g_, A*_1*g*_, and *B*_2*g *_vibration modes, respectively. The results are in good agreement with those of the rutile single crystal SnO_2 _nanowire reported previously [20]. Besides the fundamental Raman peaks, the other two Raman peaks located at about 320 and 695 cm^*-*1 ^were also observed, which correspond to IR-active *E*_*u*3_TO and *A*_2*u*_LO (TO is the mode of transverse optical phonons; LO is the mode of longitudinal optical phonons) modes, respectively [21]. The strong and sharp peak located at about 522 cm^-1 ^corresponds to the characteristic peak of the silicon substrate [22,23]. It is believed that the broadening of the peaks in the Raman scattering results is attributed to the quantum confinement effect of the sample [24].

**Figure 3:**
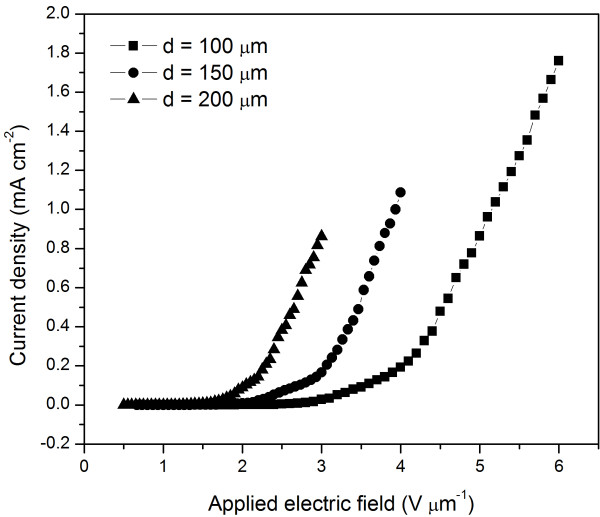
**Field-emission current density as a function of the applied electric field**. The measurements were performed at various anode-cathode separations of 100, 150, and 200 μm.

To investigate FE properties of the *in situ*-grown SnO_2 _nanowire arrays, FE measurements were performed at various anode-cathode separations. Figure 3 presents the FE current density (*J*) of the *in situ*-grown SnO_2 _nanowire arrays as a function of the applied electric field (*E*) measured at anode-cathode separations of 100, 150, and 200 μm. The turn-on field is defined as the applied electric field which produces a current density distinguished from the background noise (here, defined as 0.01 mA cm^-2^) [25]. It can be seen from the figure that the turn-on fields were dependent on the anode-cathode distance: their value decreased as the anode-cathode distance increased, and the turn-on fields were measured to be 2.8, 2.0, and 1.6 V μm^-1^, respectively. These values are lower than those reported by He et al. [26] (5.8 V μm^-1^) and Wang et al. [27] (3.77 V μm^-1^) for the SnO_2 _nanowire. The lower turn-on fields may be attributed to the good alignment of the SnO_2 _nanowire. Additionally, we believe that the *in situ *fabrication method, which made good electrical contacts between the SnO_2 _nanowire and the silicon substrate, contributed greatly to the lower turn-on fields. Moreover, the emitter radius of the SnO_2 _nanowire among the arrays was approximately 50 nm, which is small enough to make the FE performance excellent [28].

**Figure 4:**
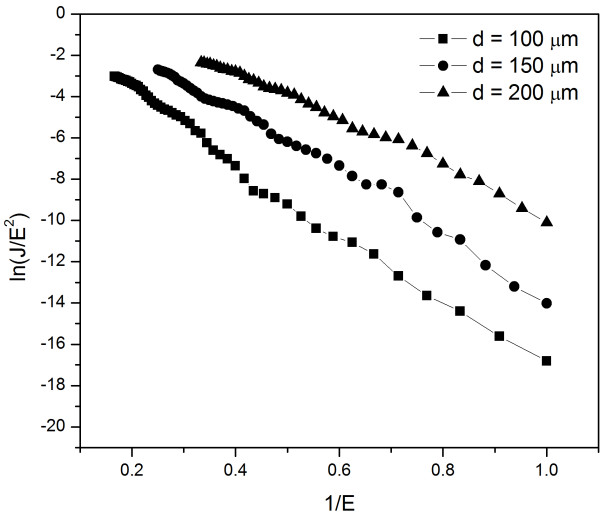
**Fowler-Nordheim plots of the field emission current densities of the *in situ*-grown SnO_2 _nanowire arrays**.

In order to understand the emission characteristics, FE properties were also analyzed by applying the classic Fowler-Nordheim [FN] law using the following equation [29]:

(1) $$J=\frac{aE_{loc}^{2}}{\varphi }\exp\left(\frac{-b\varphi^{3/2}}{E_{\text{loc}}}\right),$$

where *J *is the FE current density, *Φ *is the barrier height of the emission tip surface, and *E*_loc _is the local microscopic electric field at the emission sites. The *a *and *b *in the equation are constants with value of 1.54 × 10^-10 ^(A V^-2 ^eV) and 6.83 × 10^9 ^(V eV^-3/2 ^μm^-1^), respectively. *E*_loc_, which could be up to a hundred or thousand times of the macroscopic electric field between the cathode and anode, can be calculated using the following equation:

(2) $$E_{loc}=\beta \frac{V}{d},$$

where *β *is the field enhancement factor, *V *is the applied voltage, and *d *is the anode-cathode separation. The value of *β*, which is related to the spatial distribution of emitting centers, the crystal structure, and the geometry morphology of emitters, reflects the ability of the emitters to enhance the applied local electric field around the probe compared to the macroscopic electric field. The FN emission behavior can be evaluated from the linearity of the curves plotting ln(*J/E^2^*) versus 1/*E*. Figure 4 shows the corresponding FN plots. It can be seen that, besides the noise districts, the three plots go near to a straight line. It indicates that the field emission from the *in situ*-grown SnO_2 _nanowire arrays follows the FN relationship well, and the field emission process is a barrier tunneling quantum mechanical process [30,31].

In general, FE characteristics depend on the work function and field enhancement factor (*β*) of emitters [32]. Both density and tip morphology influence the *β *value of emitters. Ordinarily, emitters with high aspect ratios exhibit a favorable FE performance due to their much higher *β *value. The value of *β *can be calculated from the slope of a FN plot (*k*_FN_) according to the following equation:

(3) $$k=b\varphi^{3/2}\frac{d}{\beta },$$

where *φ *is the work function of SnO_2 _(4.3 eV) [33]. By analyzing the data in Figure 4, the values of *β *are estimated to be 1,082, 1,378, and 1,638 as *d *is 100, 150, and 200 μm, respectively. It can be seen that the values of *β *are high enough for practical application as field emitters.

**Figure 5:**
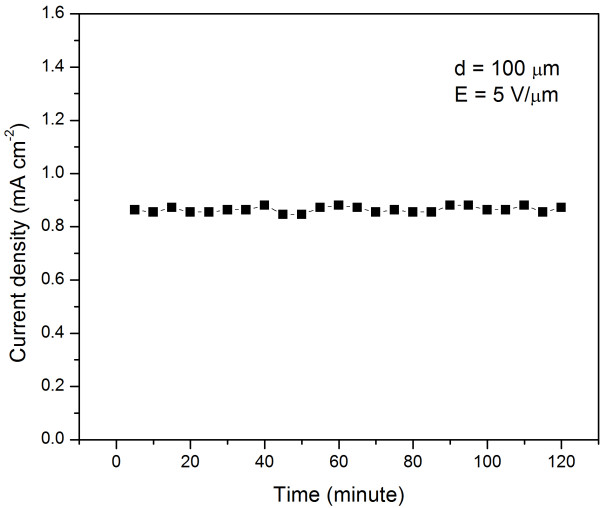
**Time dependence of the emission current of the *in situ*-grown SnO_2 _nanowire arrays**. The characterization was measured at a fixed applied voltage (500 V) with an anode-cathode separation of 100 μm.

The temporal FE current stability was measured over 120 min at an anode-cathode separation of 100 μm at a fixed voltage of 500 V. The current density fluctuation is lower than 5%, as shown in Figure 5. The stability test results confirm that the *in situ*-grown SnO_2 _nanowire arrays are competent for being high-performance field emitters [34].

To fabricate the vertically aligned SnO_2 _nanowire arrays, the thickness of a Pt catalyst is of vital importance. From many experiment results, it was found that the suitable thickness of the Pt catalyst was about 2 to 10 nm. The average length of a single SnO_2 _nanowire in the arrays can be controlled by adjusting the reaction time. It should be noted that the SnO_2 _nanowire will bend if their lengths exceeded *ca*. 100 μm due to the force of their own gravity. Additionally, it was found that no nanowire grew on a blank silicon substrate (without Pt catalyst). This feature makes the *in situ*-grown method meaningful because the emitter patterns can be well controlled and designed by selective sputtering of the Pt catalyst using traditional lithography mask technology.

## Conclusions

In summary, vertically aligned SnO_2 _nanowire arrays were deposited on a silicon substrate by an *in situ*-grown method. The FE properties of the SnO_2 _nanowire arrays were systematically studied. The FE measurement results showed that the SnO_2 _nanowire arrays had a low turn-on field of 1.6 to 2.8 V μm^-1 ^at anode-cathode separations of 100 to 200 μm. The low turn-on fields can be attributed to the vertically aligned structure and the high aspect ratio of the SnO_2 _nanowire. Moreover, the *in situ*-grown method, which makes good electrical contacts between the SnO_2 _nanowire and the silicon substrate, improved the FE performance greatly.

## Competing interests

The authors declare that they have no competing interests.

## Authors' contributions

ZHZ conducted all the experiments and drafted the manuscript. JW provided helpful guidance and suggestions. HDL helped in drafting the manuscript. ZMW supervised all of the study. All authors read and approved the final manuscript.
